# Sensitizing multi drug resistant *Staphylococcus aureus* isolated from surgical site infections to antimicrobials by efflux pump inhibitors

**DOI:** 10.4314/ahs.v20i4.16

**Published:** 2020-12

**Authors:** Amr A Baiomy, Ghada H Shaker, Hisham A Abbas

**Affiliations:** Zagazig University, Faculty of pharmacy, Department of microbiology and Immunology

**Keywords:** *Staphylococcus aureus*, multidrug resistance, efflux pump inhibitors

## Abstract

**Background:**

*Staphylococcus aureus* is a common hospital acquired infections pathogen. Multidrug-resistant Methicillin-resistant *Staphylococcus aureus* represents a major problem in Egyptian hospitals. The over-expression of efflux pumps is a main cause of multidrug resistance. The discovery of efflux pump inhibitors may help fight multidrug resistance by sensitizing bacteria to antibiotics. This study aimed to investigate the role of efflux pumps in multidrug resistance.

**Methods:**

Twenty multidrug resistant *S. aureus* isolates were selected. Efflux pumps were screened by ethidium bromide agar cartwheel method and polymerase chain reaction. The efflux pump inhibition by seven agents was tested by ethidium bromide agar cartwheel method and the effect on sensitivity to selected antimicrobials was investigated by broth microdilution method.

**Results:**

Seventy percent of isolates showed strong efflux activity, while 30% showed intermediate activity. The efflux genes *mdeA, norB, norC, norA* and *sepA* were found to play the major role in efflux, while genes *mepA, smr* and *qacA/B* had a minor role. Verapamil and metformin showed significant efflux inhibition and increased the sensitivity to tested antimicrobials, while vildagliptin, atorvastatin, domperidone, mebeverine and nifuroxazide showed no effect.

**Conclusion:**

Efflux pumps are involved in multidrug resistance in *Staphylococcus aureus*. Efflux pump inhibitors could increase the sensitivity to antimicrobials.

## Introduction

Surgical site infections (SSIs) are those infections that affect either the skin or the underlying soft tissues at the surgical site with a primarily closed incision that occurs within 30 days post-surgery 1.

Resistance of *Staphylococcus aureus* (*S. aureus*) to antimicrobial agents can take place by intrinsic or acquired resistance^2^. The emergence of multi-drug resistance may indicate the presence of efflux pumps^3^. Active efflux contributes to the antimicrobial resistance of many bacteria^4^. Bacterial efflux systems are members of larger classes of transporters responsible for the uptake of essential nutrients and ions, removal of end metabolites, harmful substances and communication between cells and the environment^5^.

In Gram-positive bacteria, efflux pumps fall into four unrelated families; small multi-drug resistance (SMR), major facilitator superfamily (MFS), adenosine triphosphate (ATP) binding cassette (ABC) and multi-drug and toxic extrusion (MATE)^6^.

To reverse efflux pump-mediated antimicrobial resistance, compounds that can inhibit efflux pumps activities; efflux pump inhibitors (EPIs) are necessary in order to treat infections caused by multidrug resistant (MDR) bacteria^7^. These compounds can be used in combination with the relevant antibiotics to achieve synergistic action. Previous studies showed that many synthetic and natural compounds can be used for this purpose. Among natural compounds, quercetin, pyrogallol and tannic acid could inhibit NorA efflux pump in *S. aureus* and the pyrogallol could potentiate gentamicin and norfloxacin against *S. aureus* 8–10. Moreover, some thiazolidinedione and thiazole derivatives were found to augment noffloxacin by interference with the activity of NorA efflux pump^11^.

The objective of this study was the detection of efflux pump as a mechanism of resistance to antimicrobials in some (MDR) *S. aureus* isolates in addition to screening for some new agents that could act as EPIs and potentiate the effect of antimicrobial agents.

## Materials and methods

### Bacterial strains

Twenty MDR *S. aureus* isolates were selected from our previous work^12^. They were obtained from patients with SSIs admitted to Surgery Department in Zagazig University Hospitals, Zagazig, Egypt.

### Media and chemicals

Mueller Hinton agar and broth, Tryptone soya agar, Nutrient agar and broth and agar were obtained from Oxoid, Hampshire, England in dehydrated form.

Domperidone, atorvastatin, nifuroxazide, sulfamethoxazole-trimethoprim, ampicillin-sulbactam and mebeverine were obtained from Egyptian Pharmaceutical Industries Company (EPICO), Egypt, while vildagliptin and metformin were obtained from National Pharmaceutical Company (NAPCO), Egypt. Imipenem as Tienam® powder for IV infusion was the marketed pharmaceutical product manufactured by Merck & Co., Inc, USA. Linezolid, levofloxacin, amikacin and doxycycline were obtained from PHARCO Company, Egypt. Clindamycin was kindly granted from PFIZER Company. PCR master mix, Agarose DNA grade, DNA marker (100 bp), Tris-acetate-EDTA (TAE) buffer (50X), verapamil, vancomycin and the PCR primers^13^ (table 1), provided by Integrated DNA Technologies were purchased from Sigma Aldrich, St. Louis, Mo, USA. DNase free water used for PCR reaction mixture was the product of Thermo Fisher Scientific, USA.

**Table 1:** Primers used in PCR amplifications

Gene	Primer	Primer sequence (5′ -3′)	Product size (bp)
*norA*	F	TTCACCAAGCCATCAAAAAG	620
	R	CTTGCCTTTCTCCAGCAATA	

*norB*	F	AGCGCGTTGTCTATCTTTCC	213
	R	GCAGGTGGTCTTGCTGATAA	

*norC*	F	AATGGGTTCTAAGCGACCAA	216
	R	ATACCTGAAGCAACGCCAAC	

*sepA*	F	GCAGTCGAGCATTTAATGGA	103
	R	ACGTTGTTGCAACTGTGTAAGA	

*mepA*	F	ATGTTGCTGCTGCTCTGTTC	718
	R	TCAACTGTCAAACGATCACG	

*mdeA*	F	AACGCGATACCAACCATTC	677
	R	TTAGCACCAGCTATTGGACCT	

*qacA/B*	F	GCTGCATTTATGACAATGTTTG	628
	R	AATCCCACCTACTAAAGCAG	

*Smr*	F	ATAAGTACTGAAGTTATTGGAAGT	285
	R	TTCCGAAAATGTTTAACGAAACTA	

bp: base pair, F: forward primer, R: reverse primer

### Methods

#### Phenotypic assessment for efflux pumps in MDR *S. aureus* isolates by ethidium bromide cartwheel method (EtBrCw) method

In order to investigate the presence of efflux pumps, the ethidium bromide cartwheel method was used. Ethidium bromide (EtBr) is an intercalating fluorescent dye that can traverse the bacterial cell wall. After entry, the dye can accumulate to a point that induces fluorescence on excitation by ultraviolet (UV) light. MDR Efflux pumps can extrude this substrate to the medium. As the efflux capacity of the cells increases, the concentration of the dye needed to produce fluorescence and the emitted fluorescence can be used to assess the efflux capacity of the isolates^14^.

Trypticase Soya Agar (TSA) plates were freshly prepared on the same day of the experiment and they contained different ethidium bromide concentrations (0.0 to 3 mg/L). The plates were kept protected from light. Each isolate was grown overnight prepared in Mueller-Hinton broth and the turbidities of the resultant suspensions were adjusted to 0.5 McFarland standard. To form a cartwheel pattern, TSA plates were divided into eight to ten sectors radially. The adjusted bacterial inocula were surface inoculated on the EtBr-TSA plates and the TSA plates were incubated at 37°C for 16 hrs. TSA plates were examined under UV transilluminator and the least concentration of EtBr that induced fluorescence of the bacterial mass was detected and the plates were photographed. Isolates that produced fluorescence at concentrations more than 2 µg/ml EtBr were considered to have positive efflux activity, while the fluorescence at concentrations less than or equal to 1 µg/ml EtBr indicated negative efflux activity. On the other hand, the presence of fluorescence at concentrations equal to 2 µg/ml EtBr indicated intermediate efflux activity 15,16.

Genotypic screening for efflux pump genes by PCR

All twenty *S. aureus* isolates were tested for the presence of six chromosomal encoded genes (norA, norB, norC, sepA, mepA, mdeA) and two plasmid encoded genes (qacA/B and smr) encoding efflux pumps in *S. aureus* by polymerase chain reaction (PCR) amplification technique^14^,^17^.

The crude cell lysate was prepared according to Nair and Venkitanarayanan^18^. Two or three colonies of overnight cultures of each isolate on nutrient agar were suspended in 50 µL PCR-quality water and then heated at 95°C for 10 minutes in PCR thermocycler. After quick centrifugation of the bacterial suspensions at 13000 rpm for 30 seconds, supernatants containing the genomic DNA were collected and stored at -20°C to be used in the PCR. Two µl of the prepared extract of each isolate was used as DNA template for PCR amplification. The amplification mixture was prepared in a final volume of 25 µL and consists of PCR Mix MyTaq ™ red Master Mix (2X) (12.5 µL, forward primer (1.5 µL), reverse primer (1.5 µL), template DNA (2 µL), nuclease-free water (7.5 µL). The DNA amplification conditions were as follows:

DNA was denatured at 94°C for 4 min, followed by 35 cycles of denaturation at 94°C for 30 s. The annealing temperatures were 60 °C for norA, 62 °C for norB and norC and 61 °C for sepA, mepA and mdeA for 45–55 s. The step of extension was carried out at 72 °C for 55 s-1 min, and was followed by a step of final extension at 72 °C for 5 min. furthermore, the PCR reactions for genes qacA/B and smr were performed under the following conditions: denaturation of DNA at 95 °C for 1 min, followed by 30 cycles of denaturation at 95 °C, each for 1 min, annealing at 58 °C for qacA/B and smr for 45 s and extension at 72 °C for 1 min, and finally a step of final extension at 72 °C for 5 min. The amplification products were electrophoresed in agarose gel (1%) for 1 h at 100 V, stained with EtBr of concentration 0.5 mg/ml^19^, and photographed under UV light.

#### Determination of minimum inhibitory concentration (MIC) of different antibiotics biocides and potential efflux pump inhibitors against *S. aureus* isolates

To determine the minimum inhibitory concentration, the broth microdilution method was used following the CLSI guidelines and Vicki al.^20^, ^21^. A suspension of four morphologically similar colonies for each isolate was made in sterile saline in order to have a turbidity matching that of 0.5 McFarland Standard. The resulting suspensions were diluted 1:100 in Mueller Hinton broth. Different two fold serially diluted antimicrobial solutions or drugs (vildagliptin, metformin, atorvastatin, domperidone, mebeverine and nifuroxazide) that were screened for their potential efflux pump inhibitory activity were made in the wells of 96-well microtiter plate so that each well contains 50µl of the antibiotic dilution. To the antibiotic-containing wells, aliquots of 50µl of suspension were transferred. The plates were incubated at 35–37°C for 18–24 hrs, and the MIC was calculated by observing the lowest concentration of the antimicrobial that inhibited visible growth of the microorganism. The antibiotics used were vancomycin, linezolid, ampicillin-sulbactam, levofloxacin, doxycycline, amikacin, sulfamethoxazole-trimethoprim, clindamycin and imipenem. The biocides used included povidone iodine, glutaraldehyde, cetrimide, chlorhexidine and crystal violet.

#### Screening for the effect of the some agents on efflux pumps activity by EtBrCw method

The cartwheel assay was used in the presence of ¼ MIC of selected drugs and the fluorescence was recorded and compared to that produced in control plates without the tested drugs. Verapamil was used as well reported control EPI, where other drugs (vildagliptin, metformin, atorvastatin, domperidone, mebeverine and nifuroxazide) were used as potential efflux pump inhibitors.

#### Effect of potential efflux pump inhibitors (EPIs) on the resistance of *S. aureus* isolates to different antimicrobial agents

In order to confirm the effect of the potential EPIs, the MICs of tested antibiotics and biocides were determined by broth microdilution method in the presence of ¼ MIC (sub-MIC) of the tested agents against *S. aureus* isolates.

## Results

### Phenotypic assessment for efflux pumps in MDR *S. aureus* isolates by EtBrCw method

**Figure 1:**
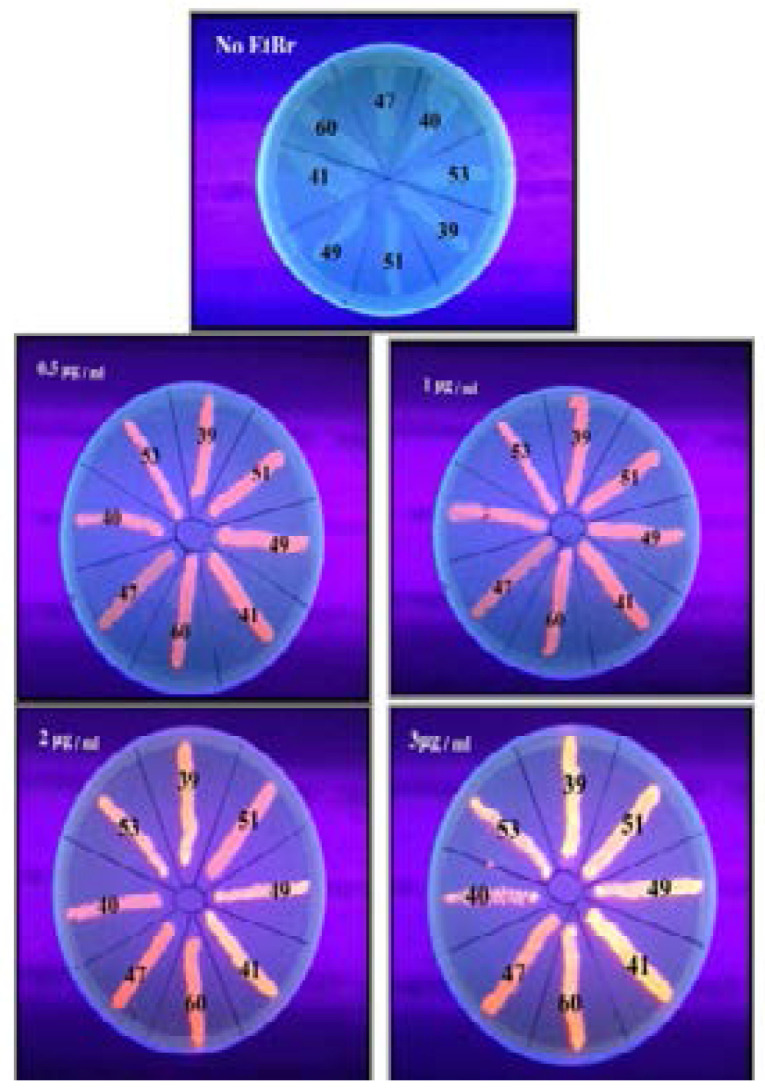
Qualitative assessment of efflux pumps activity by EtBr agar cartwheel method. Isolates that produced fluorescence at concentrations more than 2 µg/ml EtBr were considered to have positive efflux activity, while the fluorescence at concentrations less than or equal to 1 µg/ml EtBr indicated negative efflux activity. On the other hand, the presence of fluorescence at concentrations equal to 2 µg/ml EtBr indicated intermediate efflux activity. Isolates No. 39, 41, 49 and 53 showed emission of fluorescence at EtBr concentration 2µg / ml (CW intermediate), while isolates 51, 60, 47 and 40 showed no fluorescence at that concentration but showed fluorescence at 3µg / ml except isolate No. 47 which showed no fluorescence at that concentration (CW positive).

Fourteen resistant isolates (70%) were EtBrCW-positive and six isolates (30%) were EtBrCW-intermediate. The EtBrCW method was used to screen for efflux pump activity (table 2 and Fig. 1).

**Table 2:** Efflux pump activity of tested bacterial isolates by EtBr agar cartwheel method

EtBr (µg/ml) and degree of fluorescence produced					

Isolate No.	0.5(µg/ml)	1(µg/ml)	2(µg/ml)	3(µg/ml)	Efflux activity
SA 27	-	-	-	+	Positive
SA 39	-	-	+	+	Intermediate
SA 40	-	-	-	+	Positive
SA 41	-	-	+	+	Intermediate
SA 44	-	-	-	+	Positive
SA 45	-	-	-	+	Positive
SA 47	-	-	-	-	Positive
SA 49	-	-	+	+	Intermediate
SA 50	-	-	-	+	Positive
SA 51	-	-	-	+	Positive
SA 53	-	-	+	+	Intermediate
SA 60	-	-	-	+	Positive
SA 77	-	-	-	+	Positive
SA 80	-	-	-	+	Positive
SA 81	-	-	+	+	Intermediate
SA 84	-	-	-	+	Positive
SA 85	-	-	-	+	Positive
SA 87	-	-	-	-	Positive
SA 89	-	-	+	+	Intermediate
SA 90	-	-	-	+	Positive

SA: *Staphylococcus aureus*, -: No fluorescence, +: Fluorescence.

### Genotypic screening for efflux pump genes by PCR

For further identification of efflux pump as a resistance mechanism of the tested MDR *S. aureus*, the twenty MDR isolates were tested for the presence of efflux pump genes norA, norB, norC, sepA, mepA, mdeA, qacA/B and smr genes in S. aureus by PCR amplification technique.

**Figure 2:**
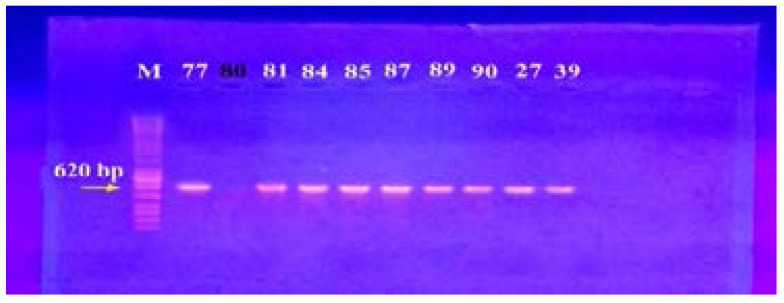
Electrophoresis of PCR products of *nor*A gene. M; marker (100 bp). Isolates 77, 81, 84, 85, 87, 89, 90, 27 and 39 showed a band at 620 bp that corresponds to *nor*A gene.

**Figure 3:**
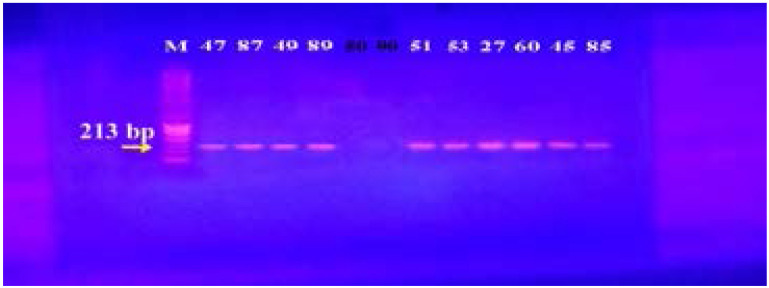
Electrophoresis of PCR products of *norB* gene. M; marker (100 bp), Isolates 47, 87, 49, 89, 51, 53, 27, 60, 45 and 85 showed a band at 213 bp, that corresponds to *norB* gene.

**Figure 4:**
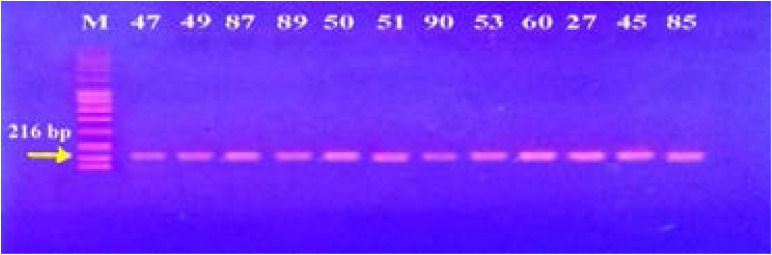
Electrophoresis of PCR products of *norC* gene. M; marker (100 bp). All isolates showed a band at 216 bp that corresponds to *norC* gene.

**Figure 5:**
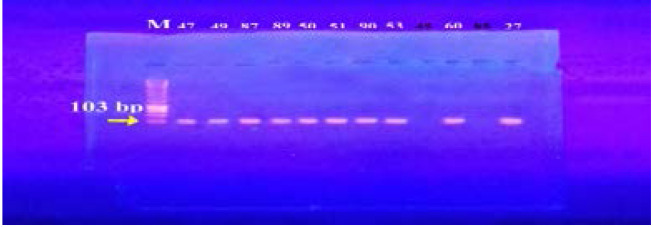
Electrophoresis of PCR products of *sep*A gene. M; marker (100 bp). Isolates 47, 49, 87, 89, 50, 51, 90, 53, 60 and 27 showed a band at 103 bp that corresponds to *sep*A gene

**Figure 6:**
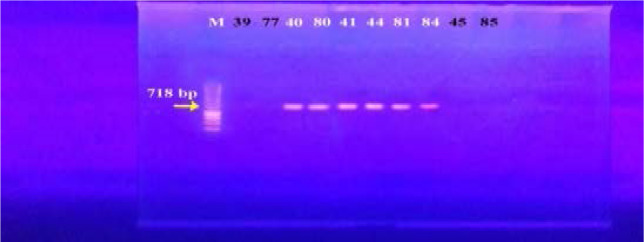
Electrophoresis of PCR products of *mep*A gene. M; marker (100 bp). Isolates 40, 80, 41, 81, 44 and 84 showed a band at 718 bp that corresponds to *mep*A gene.

**Figure 7:**
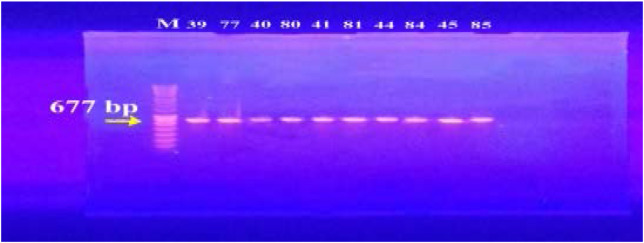
Electrophoresis of PCR products of *mdeA* gene. M; marker (100 bp). All isolates showed a band at 677 bp that corresponds to *mdeA* gene.

**Figure 8:**
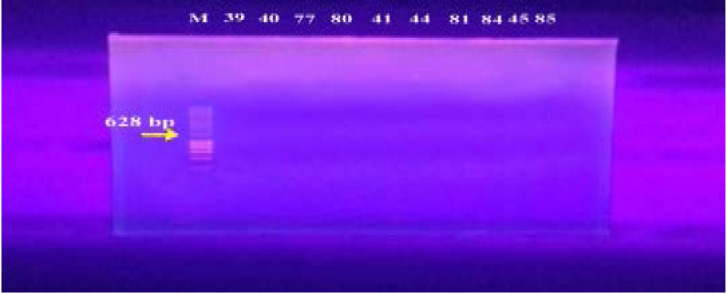
Electrophoresis of PCR products of *qacA/B* gene. M; marker (100 bp). All isolates showed no bands at 628 bp that correspond to qacA/B gene.

**Figure 9:**
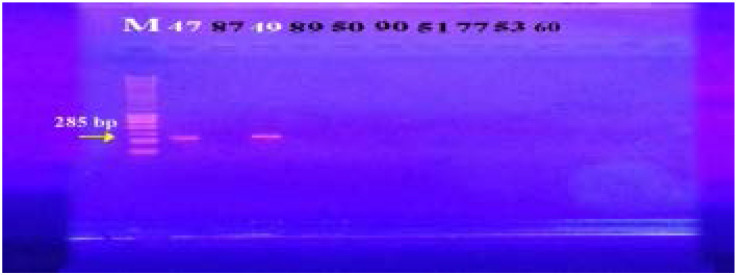
Electrophoresis of PCR products of *smr* gene. M; marker (100 bp). Isolates 47 and 49 showed a band at 285 bp that corresponds to *smr* gene.

In our study, 15 isolates (75%) were positive for norA gene (620 bp), while 18 isolates (90%) and 16 isolates (80%) showed norB (213 bp) and norC (216 bp) genes, respectively (Figs. 2–4). Moreover, sepA gene (103 bp) was found in 16 isolates (80%). On the other hand, mepA gene (718 bp) was present in 8 isolates (40%) (Figs. 5–6). Furthermore, mdeA gene (677 bp)was found in 18 isolates (90%) (Fig. 7), while qacA/B gene (628 bp)was absent in all isolates and smr gene (285 bp) was found in only 2 isolates (10%) (Figs. 8–9).

The most prevalent genes found in the isolates were the six chromosomal genes (mdeA, norB, norC, sepA, norA, mepA), that were found in 40–90% of the isolates, while the two plasmid genes (qacA/B and smr) were the least prevalent ones among the tested isolates (0–10% of the isolates).

Assessment the effect of tested agents on the resistance of multi-drug resistant bacteria for efflux pumps by ethidium bromide agar cartwheel method

**Figure 10:**
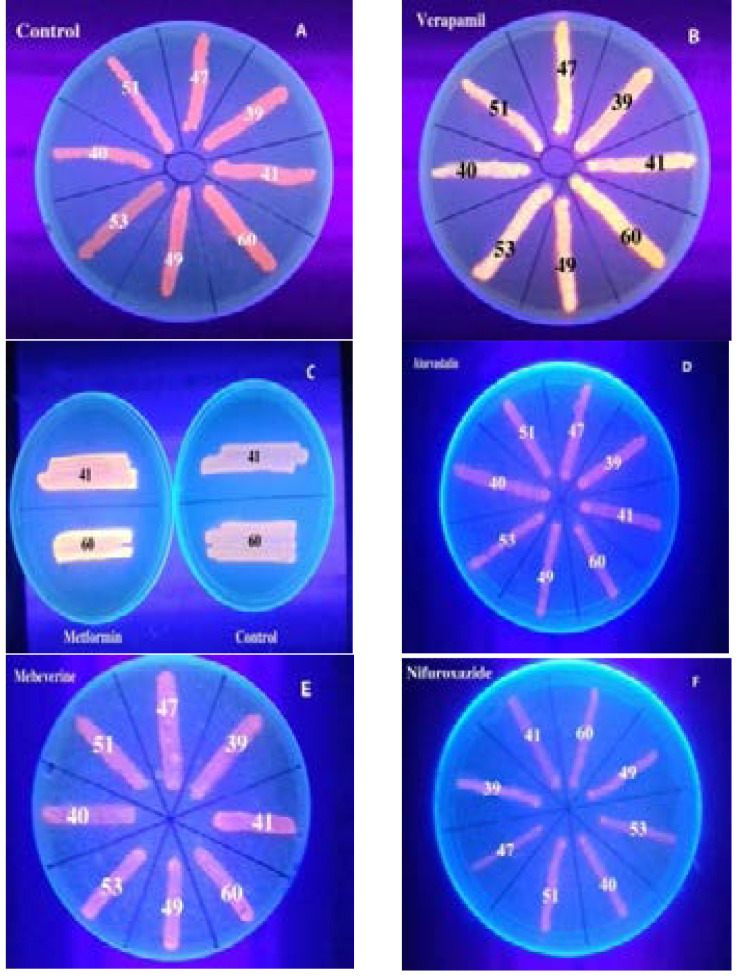
Effect of the tested agents on efflux pump by EtBrCW method A) Control plate with no drugs, B) Verapamil and C) Metformin enhanced the fluorescence indicating a possible EPI activity, D) Atorvastatin, E) Mebeverine and F) Nifuroxazide had no effect on fluorescence indicating no effect on efflux pump activity.

The cartwheel assay was used in the presence and absence of sub-MIC of selected drugs and the fluorescence was compared. Verapamil as a well reported efflux inhibitor increased the fluorescence indicating its effect on inhibition of efflux pumps, while mebeverine, vildagliptine, domperidone, nifuroxazide and atorvastatin showed no effect. Metformin was tested as efflux pump inhibitor in 2 MDR isolates and it enhanced the fluorescence indicating a possible EPI activity (Fig. 10).

### Effect of potential EPIs on the resistance of *S. aureus* isolates to different antimicrobial agents

In order to confirm the results of EtBrCw method, the MICs of tested antibiotics and biocides were determined by broth microdilution method against *S. aureus* isolates in the presence of ¼ MIC (sub-MIC) of the tested agents that were positive for EtBrCw method (verapamil and metformin). The minimum inhibitory concentrations of the tested drugs were 0.25 mg/ml for nfuroxazide, 1 mg/ml for verapamil, mebeverine and atorvastatin, 8 mg/ml for vildagliptin and 32 mg/ml for each of metformin and domperidone. Sub-MICs of verapamil and metformin significantly reduced the MICs of antibiotics and biocides. Metformin showed much higher activity in reducing MICs (64-2048 fold decrease) as compared with the well-known efflux pump inhibitor verapamil (2–512 fold decrease) as shown in tables (3–6).

**Table 3:** Synergistic activity of verapamil with antimicrobial agents against MDR *S. aureus* isolates

Isolate No.	MIC in µg/ml in the absence (presence) of verapamil													

	LZD	SAM	LEV	AK	VA	SXT	DO	DA	IMP	CET	GLU	CHX	CV	PI
SA 27	8(0.5)	8/4 (0.016/0.008)	4 (0.25)	64 (8)	0.25 (0.03)	4\76 (0.5/9.5)	4 (1)	1 (0.06)	1 (0.125)	4 (0.5)	5000 (1250)	0.5 (0.03)	4 (1)	5000 (312.5)
SA 39	4(0.06)	8/4 (0.125/0.06)	16 (1)	64 (1)	2 (0.06)	2\38 (0.03/0.59)	64 (4)	1024 (64)	4 (1)	32 (4)	2500 (156.25)	0.5 (0.06)	4 (0.5)	5000 (312.5)
SA 40	8(0.5)	32/16 (2/1)	4 (0.25)	16 (0.25)	8 (0.06)	4\76 (0.06/1.19)	2 (0.06)	16 (1)	2 (0.25)	2 (0.125)	2500 (625)	0.5 (0.125)	4 (1)	2500 (312.5)
SA 41	2(0.03)	64/32 (2/1)	16(2)	64(1)	4 (1)	4\76 (0.125/2.38)	1 (0.25)	16 (8)	0.125 (0.06)	8 (0.5)	5000 (625)	1 (0.03)	4 (0.5)	2500 (156.25)
SA 44	1(0.03)	128/64 (1/0.5)	4 (0.25)	64 (0.5)	4 (0.125)	4\76 (0.03/0.59)	16 (4)	1024 (32)	2 (0.06)	4 (0.25)	2500 (312.5)	0.5 (0.125)	4 (1)	5000 (625)
SA 45	4(0.25)	32/16 (1/0.5)	1 (0.06)	64 (16)	32 (1)	8\152 (2/38)	4 (0.5)	8 (2)	16 (0.5)	8 (1)	5000 (1250)	0.5 (0.06)	4 (0.5)	1250 (78.25)
SA 47	16(0.5)	8/4 (4/2)	4 (1)	64 (1)	128 (4)	4\76 (0.06/1.19)	32 (4)	1024 (32)	4 (1)	32 (8)	2500 (156.25)	4 (1)	4 (1)	2500 (312.5)
SA 49	2(1)	32/16 (1/0.5)	16 (1)	128 (16)	4 (2)	8\152 (1/19)	32 (2)	16 (1)	32 (8)	4 (0.5)	2500 (312.5)	1 (0.25)	4 (1)	2500 (156.25)
SA 50	256(0.5)	128/64 (2/1)	16 (0.5)	128 (32)	512 (2)	4\76 (0.06/1.19)	16 (1)	1024 (128)	0.25 (0.03)	4 (0.5)	2500 (312.5)	1 (0.25)	4 (1)	2500 (156.25)
SA 51	4(0.5)	32/16 (1/0.5)	8 (1)	16 (0.25)	1 (0.125)	2\38 (0.125/2.38)	32 (2)	1024 (16)	0.125 (0.03)	4 (0.5)	5000 (312.5)	0.5 (0.03)	4 (0.5)	2500 (156.25)
SA 53	8(0.25)	32/16 (1/0.5)	8 (0.25)	16 (0.25)	512 (8)	4\76 (0.06/1.19)	4 (1)	16 (8)	16 (2)	2 (0.125)	5000 (625)	0.5 (0.06)	4 (0.5)	2500 (156.25)
SA 60	1(0.06)	16/8 (0.031/0.016)	4 (0.125)	64 (2)	32 (1)	2\38 (0.06/1.19)	16 (1)	128 (16)	2 (0.25)	2 (0.25)	10000 (1250)	0.5 (0.125)	2 (0.5)	2500 (312.5)
SA 77	8(0.25)	8/4 (0.25/0.125)	4 (0.25)	128 (16)	0.25 (0.06)	4\76 (0.5/9.5)	32 (16)	16 (2)	1 (0.25)	4 (0.25)	5000 (312.5)	0.5 (0.03)	2 (0.5)	5000 (156.25)
SA 80	8(0.25)	32/16 (1/0.5)	8 (0.25)	16 (0.25)	8 (0.125)	4\76 (0.06/1.19)	16 (1)	16 (1)	2 (1)	4 (0.5)	5000 (1250)	0.5 (0.06)	1 (0.125)	5000 (312.5)
SA 81	1(0.06)	64/32 (4/2)	16 (8)	64 (2)	4 (1)	4\76 (0.06/1.19)	1 (0.25)	32 (1)	0.125 (0.016)	4 (0.5)	2500 (156.25)	0.5 (0.06)	1 (0.125)	5000 (625)
SA 84	1 (0.03)	128/64 (2/0.5)	16 (1)	64 (0.5)	1 (0.0.6)	4\76 (0.03/0.59)	1\4 (0.016)	1024 (64)	2 (0.25)	4 (0.25)	10000 (625)	0.5 (0.03)	4 (1)	2500 (156.25)
SA 85	4 (0.25)	32/16 (0.5/0.25)	1(0.03)	128 (32)	64 (2)	8\152 (4/76)	4 (0.5)	32 (4)	16	2 (0.125)	5000 (312.5)	0.5 (0.06)	4 (1)	2500 (156.25)
SA 87	32 (1)	8/4 (0.016/0.008)	4 (0.25)	64 (1)	128 (0.125)	4\76 (0.06/1.19)	8 (1)	512 (16)	4	2 (0.25)	5000 (625)	0.5 (0.06)	4 (1)	5000 (312.5)
SA 89	1 (0.03)	32/16 (0.5/0.25)	64 (2)	128 (16)	4 (2)	8\152 (1/19)	32 (1)	16 (1)	32	8 (1)	5000 (312.5)	0.5 (0.125)	2 (0.25)	5000 (625)
SA 90	64 90.25)	128/64 (2/1)	16 (4)	128 (64)	512 (2)	4\76 (0.06/1.19)	16 (1)	64 (16)	1\4	4 (0.25)	5000 (625)	0.5 (0.125)	2 (0.5)	5000 (625)

SA: *Staphylococcus aureus*, LZD: Linzeolid, SAM: Ampicillin-sulbactam, LEV: Levofloxacin, AK: Amikacin, VA: Vancomycin, SXT: Sulfamethoxazole-trimethoprim, DO: Doxycycline, DA: Clindamycin, IPM: Imipenem, CET: Cetrimide; GLU: Glutaraldehyde, CHX: Chlorhexidine; CV: Crystal violet, PI: Povidone-iodine.

**Table 4:** Effect of verapamil on susceptibility of *S. aureus* to antimicrobial agents

Isolate No.	Folds decrease in MIC													

	LZD	SAM	LEV	AK	VA	SXT	DO	DA	IMP	CET	GLU	CHX	CV	PI
SA 27	16	512	16	8	8	8	4	16	8	8	4	16	4	16
SA 39	64	64	16	64	32	64	16	16	4	8	16	8	8	16
SA 40	16	16	16	64	128	64	32	16	8	16	4	4	4	8
SA 41	64	32	8	64	4	32	4	2	2	16	8	16	8	16
SA 44	32	128	16	128	32	128	4	32	32	16	8	4	4	8
SA 45	16	32	16	4	32	4	8	4	32	8	4	8	8	16
SA 47	32	2	4	64	32	64	8	32	4	4	8	4	4	8
SA 49	2	32	16	8	2	8	16	16	4	8	16	4	4	16
SA 50	512	64	32	4	256	64	16	8	8	8	8	4	4	16
SA 51	8	32	8	64	8	16	16	64	4	8	8	16	8	16
SA 53	32	32	32	64	64	64	4	2	32	16	16	8	8	16
SA 60	32	512	32	32	32	32	16	8	8	8	8	4	4	8
SA 77	16	32	16	8	4	8	2	8	4	16	8	16	4	32
SA 80	16	32	32	64	128	64	16	16	2	8	16	8	8	16
SA 81	32	16	2	32	4	64	4	32	8	8	4	8	8	8
SA 84	32	64	16	128	16	128	16	16	8	16	16	16	4	16
SA 85	16	64	32	4	32	2	8	8	16	16	16	8	4	16
SA 87	32	512	16	64	8	64	8	32	4	8	8	8	4	16
SA 89	32	64	32	8	2	8	32	16	4	8	16	4	8	8
SA 90	16	64	4	2	256	64	16	4	16	16	8	4	4	8

SA: *Staphylococcus aureus*, LZD: Linzeolid, SAM: Ampicillin-sulbactam, LEV: Levofloxacin, AK: Amikacin, VA: Vancomycin, SXT: Sulfamethoxazole-trimethoprim, DO: Doxycycline, DA: Clindamycin, IPM: Imipenem, CET: Cetrimide; GLU: Glutaraldehyde, CHX: Chlorhexidine; CV: Crystal violet, PI: Povidone-iodine.

**Table 5:** Synergistic activity of metformin with antimicrobial agents against representative MDR *S. aureus* isolates

Isolate No.	MIC in µg/ml in the absence (presence) of verapamil													

	LZD	SAM	LEV	AK	VA	SXT	DO	DA	IMP	CET	GLU	CHX	CV	PI
SA 41	2 (0.002)	64/32 (0.06/0.03)	16 (0.016)	64 (0.03)	4 (0.004)	4\76 (0.004/0.074)	1 (0.001)	16 (0.008)	0.125 (0.0001)	8 (0.06)	5000 (78.125)	1 (0.002)	4 (0.06)	2500 (19.53)
SA 60	1 (0.001)	16/8 (0.016/0.008)	4 (0.002)	64 (0.06)	32 (0.016)	2\38 (0.008/0.037)	16 (0.016)	128 (0.125)	2 (0.002)	2 (0.016)	10000 (39.06)	0.5 (0.008)	2 (0.08)	2500 (39.06)

SA: *Staphylococcus aureus*, LZD: Linzeolid, SAM: Ampicillin-sulbactam, LEV: Levofloxacin, AK: Amikacin, VA: Vancomycin, SXT: Sulfamethoxazole-trimethoprim, DO: Doxycycline, DA: Clindamycin, IPM: Imipenem, CET: Cetrimide; GLU: Glutaraldehyde, CHX: Chlorhexidine; CV: Crystal violet, PI: Povidone-iodine.

**Table 6:** Effect of metformin on susceptibility of *S. aureus* to antimicrobial agents

Isolate No.	Fold decrease in MIC													
	LZD	SAM	LEV	AK	VA	SXT	DO	DA	IMP	CV	CET	GLU	CHX	PI
SA 41	1024	1024	1024	2048	1024	1024	1024	2048	1024	64	128	64	512	128
SA 60	2048	1024	2048	1024	2048	1024	1024	1024	1024	256	128	256	64	64

SA: *Staphylococcus aureus*, LZD: Linzeolid, SAM: Ampicillin-sulbactam, LEV: Levofloxacin, AK: Amikacin, VA: Vancomycin, SXT: Sulfamethoxazole-trimethoprim, DO: Doxycycline, DA: Clindamycin, IPM: Imipenem, CET: Cetrimide; GLU: Glutaraldehyde, CHX: Chlorhexidine; CV: Crystal violet, PI: Povidone-iodine.

## Discussion

The global crisis of the emergence of antibiotic resistance among bacteria, especially multidrug resistance (MDR) made it urgent to discover new antibacterials and agents that can play as resistance modifiers (EPIs). Efflux pumps have the ability to expel structurally unrelated compounds, including antibiotics used in a clinical setting and hence antibiotics lost their clinical efficacy. The over-expression of efflux pumps can lead to rapid development of antibiotic resistance. This can take place even to the antibiotics of last resort. It is therefore necessary to investigate the ability of new drugs to act as efflux pump inhibitors (EPIs). By the introduction of EPIs, the traditional therapeuticaly ineffective antibiotics could find its way back to clinical use^22^, ^23^.

Efflux systems in *Staph. aureus* are critical for exporting antibiotics and detergents^4^. Therefore, the selected MDR isolates in this study were screened phenotypically for efflux pumps by EtBrCw method and genotypically by PCR.

Active efflux was detected in all isolates by the cartwheel assay. This was in accordance with Rana et al.^24^ in which all MDR *Staphylococcal* isolates showed active efflux.

In order to confirm phenotypic study of efflux pump as a resistance mechanism, genotypic investigation of efflux pump genes was done. PCR amplification technique was performed to detect the prevalence of norA, norB, norC, sepA, mepA, mdeA, qacA/B and smr geneslux mediated resistance^8^.

The study revealed that mdeA and norB genes were the most frequent ones (90%), while qacA/B gene was absent in all isolates. This was more or less similar to the results reported in Iran by Sepideh et al.^13^, in which the most prevalent gene was mdeA (61.7%), while norB gene was present in 41.7% of isolates. Moreover, qacA/B gene was present in only 3.3% of isolates. Furthermore, a canadian report stated that the distribution of qacA/B was only 2% 25. In contrast, our results were much lower than an Asian study that reported prevalence rates of 73% for the qacA/B26. Moreover, the prevalence rates of norA, norC, sepA and mepA genes were 75%, 80%, 80% and 40%, respectively. However, the study of Sepideh et al.13 reported lower prevalence rates; 41.7%, for both of norA and norC genes and 35% for sepA, while the rate of presence of mepA was higher than this study (60%).

Smr gene was found in 10% of isolates only. These results were in agreement with that obtained by Longtin et al.^25^ in Canada that reported prevalence rate of 7%. In contrast, these results were lower than a study in Asia that reported prevalence rate of 32% for smr gene^26^.

From the obtained results it might be concluded that genes mdeA, norB, norC, norA and sepA play the major role in efflux, while genes mepA, smr and qacA/B play a minor role in efflux. Strong correlation between active efflux as a resistance mechanism and antimicrobials MDR found in this study was supported by Costa et al. 14, who reported that active efflux is a major mechanism in the early stages of resistance development. The variation in the frequency of efflux genes among different studies may be due to temporal and geographical variation^27^.

To combat efflux-mediated resistance, natural or synthetic compounds were investigated in previous studies and many compounds were proved to have inhibitory activities against efflux pumps in *S. aureus*. The TetK efflux-pump in *S. aureus* was blocked by the essential oil of *Chenopodium ambrosioides L*. and its α-terpinene active ingredient^28^. Moreover, the natural compound Juglone could reverse the efflux-mediated resistance of *S. aureus* to tetracycline, erythromycin and benzalkonium chloride^29^. Some synthetic amino acid amides of piperic acid and 4-ethylpiperic acid were reported to have efflux inhibiting activity in S. aureus and when used in combination with ciprofloxacin, synergism was found^30^.

In our study, some FDA approved drugs such as verapamil, metformin, mebeverine, domperidone, vildagliptin, nifuroxazide and atorvastatin were tested for their potential efflux pump inhibiting activities. Mebeverine, domperidone, vildagliptin, nifuroxazide and atorvastatin showed no effect on the fluorescence of EtBr indicating no EPI activity upon screening by the cartwheel method. On the other hand, verapamil and metformin enhanced the fluorescence indicating their effects on inhibition of efflux pumps. Verapamil as well reported EPI causes accumulation of EtBr within the cells and induce fluorescence at a much lower concentration of EtBr. Metformin was reported as an effective inhibitor of P-glycoprotein expression and MDR reversing agent^31^. Metformin can decrease the consumption of oxygen in mitochondria and alter the distribution of charge in addition to the membrane charge. Furthermore, it was found to interfere with the transport of protons and other cations across membranes. Metformin was found beneficial in preventing breast cancer cell growth through augmenting the cytotoxicity of chemotherapeutic agents in tumor cells and reducing the high levels of MDR1 mRNA and protein in these cells^32^.

In order to confirm the effect of the well reported EPI verapamil, and the suggested one metformin on efflux mechanism in the selected MDR isolates, the MICs of antibiotics and biocides were detected in the presence and absence of sub-inhibitory concentrations of the drugs. In general, the tested agents caused significant reduction in MICs with variable degrees. In order to screen the effect of EPIs within these ranges, fold decrease in MICs exhibited by 50% of isolates was calculated to compare the potency of EPIs.

Metformin was more potent than verapamil. Metformin reduced MICs of all tested antibiotics by 1024–2048 folds. Additionally, it reduced MICs of all tested biocides by 64–512 folds. Moreover, verapamil reduced the MICs of β-lactams, vancomycin and linezolid by 32 folds. Moreover, it reduced the MICs of levofloxacin, clindamycin and doxycycline by 16 folds and MICs of amikacin and sulfamethoxazole-trimethoprim by 64 folds. Additionally, it reduced the MICs of povidone iodine, cetrimide, glutaraldehyde and chlorhexidine by 8 folds and crystal violet by 4 folds. These findings were supported by El-Domany^33^, who reported that combination of verapamil at sub MICs with β-lactams, fluoroquinolones and tetracyclines resulted in sharp reduction in the MICs ranged between 8 and 128 folds. Also, our findings were supported by Gabr^34^, who found that the MICs decrease with the use of verapamil ranged between 8–64-folds for β-lactams, 4–32 folds for ciprofloxacin, 8–32-folds for macrolides, gentamicin and tetracycline. Additionally, Gabr34 found that MICs decrease with the use of verapamil were 4–16 folds for povidone iodine, cetrimide and chlorhexidine in accordance with our results.

A study conducted by Abbas^35^ revealed that the MICs of tested agents (azithromycin, crystal violet, ciprofloxacin, cefoperazone, tetracycline and streptomycin) were reduced up to 64 folds in the presence of verapamil. This sharp reduction in MICs of antimicrobials upon combination with the efflux inhibitors suggested presence of efflux pumps that extrude these antimicrobials out of the cells of multi-drug resistant isolates. This may reveal that efflux activity has a strong involvement in the reduced susceptibility to antimicrobials^13^.

## Conclusion

It could be concluded that active efflux plays a great role in multi-drug resistance as investigated phenotypically and genotypically. This study also highlighted the positive significant effect of EPIs to combat the high multi-drug resistance to antimicrobial.

Further work is needed to confirm the effect of efflux inhibitors on the expression of efflux pump genes by realtime PCR and to investigate their effects in vivo.
